# Association of Paraspinal Muscle CSA and PDFF Measurements With Lumbar Intervertebral Disk Degeneration in Patients With Chronic Low Back Pain

**DOI:** 10.3389/fendo.2022.792819

**Published:** 2022-05-26

**Authors:** Yilong Huang, Ling Wang, Xiaomin Zeng, Jiaxin Chen, Zhenguang Zhang, Yuanming Jiang, Lisha Nie, Xiaoguang Cheng, Bo He

**Affiliations:** ^1^ Department of Medical Imaging, the First Affiliated Hospital of Kunming Medical University, Kunming, China; ^2^ Department of Radiology, Beijing Jishuitan Hospital, Beijing, China; ^3^ GE Healthcare, Magnetic Resonance Field Application Team, Chengdu, China; ^4^ GE Healthcare, Magnetic Resonance Research China, Beijing, China

**Keywords:** chronic low back pain, quantitative MRI, paraspinal muscles, fatty infiltration, lumbar intervertebral disk degeneration

## Abstract

There is an interaction between the lumbar spine and paraspinal muscles, which may play a role in the development of intervertebral disc (IVD) degeneration and may affect CLBP. The study aims to assess the relationship between IVD degeneration and paraspinal muscle fat infiltration in CLBP patients by quantitative MR imaging, and to evaluate the influence of sex and age on CLBP muscle fat infiltration. Sixty CLBP patients (46.3 years ±17.0) and thirty-two healthy subjects (44.9 years ±17.6) were recruited for this study. 3.0 T MRI was used to perform the sagittal and axial T1, T2 of the lumbar spine, and axial paraspinal muscle IDEAL imaging at the L4/5 and L5/S1 levels. Proton density fat fraction (PDFF) of the multifidus and erector spinae at two IVD levels were measured. The Pfirrmann grades of IVD degeneration, Oswestry Disability Index (ODI), and Visual Analog Scale (VAS) were also evaluated. Compare the cross-sectional area (CSA) and PDFF of the paraspinal muscles between CLBP patients and healthy subjects, and analyze the relationship between the muscle PDFF and Pfirrmann grades, gender, and age of CLBP patients. Compared with healthy subjects, the CSA of the multifidus muscle in CLBP patients decreased (1320.2±188.1mm^2^ *vs.* 1228.7±191.0 mm^2^, *p*<0.05) at the L4/5 level, the average PDFF increased, (7.7±2.6% *vs.* 14.79±5.3%, 8.8±4.2% *vs.* 16.03±5.3%, all *p*<0.05) at both L4/5 and L5/S1 levels. The PDFF of paraspinal muscles were correlated with adjacent IVD degeneration, ODI and VSA in CLBP patients (all *p*<0.05). After using age and body mass index (BMI) as control variables, significance was retained (all p<0.05). Multiple regression analysis revealed sex and age also were significantly associated with multifidus PDFF (all *p* < 0.05). This study confirmed that the CSA decreased and the PDFF increased of the paraspinal muscles in CLBP patients. It reveals a significant correlation between the PDFF of CLBP paraspinal muscles and the grade of IVD degeneration. Sex and age are also important factors influencing CLBP paraspinal muscle infiltration.

## Introduction

Low back pain (LBP) has become a global challenge with tremendous economic burden for society and public health systems (1, 2). The lifetime prevalence of LBP is reported to be as high as 84%, with chronic low back pain (CLBP) accounting for approximately 23% of LBP (3). Furthermore, more than 10% of patients with LBP develop severe disabilities (4). The diversity and complexity of etiology limit the prevention and treatment strategies of LBP. Intervertebral disc (IVD) degeneration refers to the physiological and pathological process of natural degeneration and aging of the IVD, in which structural damage causes the degeneration of the disc and the surrounding area (5). IVD degeneration is the basis of various clinical spinal diseases, for example, annulus tears, instability of the spine, degeneration in the facet joints, disc herniation, spinal stenosis and CLBP (5, 6). And IVD degeneration is usually considered as the leading cause of CLBP, especially at the L4-S1 level, but the treatments are mainly limited to partial symptomatic relief (7, 8).

The paraspinal muscles (multifidus, erector spinae, and psoas) are essential determinants of the structural stability and functions of the lumbar spine (9). Previous animal and human studies suggested that increased myoelectric activity and structural remodeling of muscles (e.g. muscle atrophy, fat infiltration, and fiber type changes) were associated with CLBP (10–14). Given the important role of paraspinal muscles on the lumbar spine, muscle lesions may worsen CLBP. It is crucial to study the interactions between paraspinal muscle changes and CLBP, but they are often underestimated. In addition, it is unclear whether the degeneration of the lumbar IVD is related to increased fatty infiltration within the paraspinal muscles in CLBP patients.

Previous studies had reported the muscle cross-sectional area (CSA) and fat content of the paraspinal muscles in CLBP patients (15–18). Based on anatomical imaging, the CSA variable of muscles is routinely preferred (19), which can be used as a structural measure of muscle hypertrophy or atrophy. The assessments of fat infiltration were mainly based on the decrease of CT attenuation values (14–16, 20, 21) or the increase of the relative signal intensity on the conventional MRI T1 and T2 images (17, 22, 23). There is no published literature on CLBP related to the intramuscular adipose tissue in CLBP study. In recent years, an advanced chemical shift encoding-based water-fat MRI has been used for non-invasive quantitative assessment of fat and water signals in various parts of the human body (24–26), such as available Iterative Decomposition of water and fat with Echo Asymmetry and Least Square Estimation (IDEAL-IQ). Proton density fat fraction (PDFF) outcomes can be obtained with high resolution and high accuracy from IDEAL-IQ (25). This method is considered to be a reliable measurement method comparable to MR spectroscopy (the gold standard method *in vivo*) for quantifying fat infiltration in muscles (27–29). Sollmann et al. showed that the PDFF measurement after paraspinal muscle segmentation is a potential biomarker for muscle changes in the future (30). Furthermore, Zhao and Patzelt et al. found a negative correlation between the PDFF of paraspinal muscles and the bone mineral density of the lumbar spine, and the progress and severity of tumor cachexia can be monitored through the PDFF of the paraspinal muscles (31, 32). Therefore, PDFF help accurately quantify the fat content, especially intramuscular lipids in the paraspinal muscles of CLBP, and further explore the relationship between IVD degeneration and paraspinal muscle remodeling in CLBP patients. Furthermore, results of previous studies reveal that a decrease in the multifidus CSA, a decrease of muscle density, and a decrease in the size of Type I and Type II/MHC-2X fibers and interstitial fibrosis in patients with intervertebral herniation. And the fat infiltration may be associated with these muscle changes (14, 33, 34). We hypothesized that paraspinal muscle CSA and fat content are changed in CLBP patients compared to healthy subjects, significantly, and are associated with the degeneration of the adjacent IVD.

The purpose of our study was to compare the CSA and PDFF of paraspinal muscles in patients with CLBP and healthy subjects using novel quantitative MRI, investigate the relationship between IVD degeneration and paraspinal muscle fat infiltration. Furthermore, we compared the age-related and sex-related changes in CSA and PDFF of the paraspinal muscles in patients with CLBP.

## Materials and Methods

### Participants

In this retrospective study, sixty patients with CLBP and thirty-two healthy subjects were selected in this study from January 2019 to December 2020 (46 males, 46 females; mean age: 45.82 years; age range: 24-72 years). Patients with CLBP and healthy subjects were matched for age and sex. Informed consent forms were signed by each participant, and ethical committee approval was obtained. The inclusion criteria were as follows: the untreated patient has symptoms of LBP for more than 3 months; Healthy subjects have no symptoms of LBP;BMI ranged from 18.5 to 23.9 kg/m^2^. The exclusion criteria were visceral LBP (such as urinary tract stones); spinal trauma, fracture, tumor, infection, deformity, spondylolisthesis, surgery, and other musculoskeletal diseases; pregnancy; and contraindications for MRI. Except that the subjects in the control group had no low back pain, the other inclusion and exclusion criteria were the same as those in the low back pain group. The control group did not have any clinical symptoms, and the other inclusion and exclusion criteria were identical to those described previously. The selected subjects also completed the Oswestry Disability Index (ODI) (35) and Visual Analog Scale (VAS) (36) to assess the level of back pain and dysfunction. The ODI covers 10 items (pain, lifting, walking, social life, personal care, sitting, standing, sleeping, travelling and sex life), and each scored from 0 to 5. Total ODI score = score of each item × 2, the total ODI score ranges from 0 to 100. A higher total ODI score reflects higher disability. According to the median age of the included participants, participants under 45 years old are classified as the young group, and 45 years old or older are classified as the elderly group. **Table 1** shows the baseline clinical characteristics of the participants.

**Table 1:** Comparison of clinical characteristics and CSA of paraspinal muscle between healthy subjects and CLBP patients.

Characteristics	Healthy subjects (n = 32)	CLBP (n = 60)	*p*
Male/Female	16/16	30/30	1.000
Age (year)	44.87 ± 17.59	46.32 ± 16.99	-0.412
Male	44.99 ± 16.51	45.80 ± 15.77	-0.350
Female	44.75 ± 18.27	47.21 ± 17.69	-0.650
Height (cm)	164.13 ± 3.01	162.65 ± 3.28	0.069
Weight (kg)	58.50 ± 3.01	57.76 ± 4.71	0.110
BMI (kg/m^2^)	21.75 ± 1.23	21.71 ± 1.16	0.056
Male	22.20 ± 1.14	22.34 ± 1.22	0.077
Female	21.30 ± 1.25	21.08 ± 1.08	0.054
CSA of MF (mm^2^)			
L4/5	1320.22 ± 188.10	1228.72 ± 190.99	0.041^*^
L5/S1	1271.01 ± 213.39	1127.48 ± 282.89	0.720
CSA of ES (mm^2^)			
L4/5	2320.40 ± 303.77	2263.16 ± 485.71	0.398
L5/S1	1127.48 ± 282.89	999.04 ± 463.23	0.907
ODI scores	NA	28.45 ± 13.17	–
VAS scores	NA	5.99 ± 1.42	–

CLBP, chronic low back pain; CSA, cross-sectional area; MF, multifidus; ES, erector spinae. ODI, Oswestry Disability Index; VAS, Visual Analog Scale. All values were expressed as mean ± standard deviation. Significant p-values are marked with “*”. NA, Not Applicable.

### MR Data Acquisition

All MRI experiments were performed using a 3.0T MR system (Discovery 750w, GE Healthcare, USA). A 32-channel the phased array spine coil was used for CLBP patients and healthy subjects. To reduce motion artifacts, an abdominal bandage was used to compress the abdomen and a wedge-shaped foam pad was placed under the lower limbs of participants in a standard supine position. MRI scanning for participants included sagittal T1-weighted imaging (T1), T2-weighted imaging (T2) of the lumbar spine, and axial T2, IDEAL-IQ of paraspinal muscles. The MRI protocols of participants are summarized in **Table 2**.

**Table 2:** MRI scan parameters.

Images	TE	TR	ST	SL	FOV	NEX	Spatial resolution (mm^2^)	Acquisition time
	(ms)	(ms)	(mm)	(mm)	(mm^2^)			
Sagittal T1	8.3	361	4	4	320×180	2	1.0×1.4	1 min 28 s
Sagittal T2	142	2500	4	4	320×180	2	1.0×1.4	1 min 25 s
Axial T2	110.5	4024	3	4	200×200	3	0.7×0.9	1 min 15 s
IDEAL-IQ	1.2/3.2/5.2/ 7.2/9.2/11.2	7.8	4	0	240×240	2	1.0×1.5	5 min 35 s

TR, time of repetition; TE, echo time; ST, slice thickness; SL, slice increment; FOV, field of view; NEX, number of excitation.

### Image Analyses

**Figure 1:**
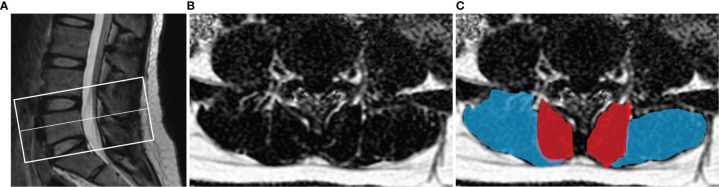
Demonstration of paraspinal muscle segmentation. **(A)** The center level of the scan was at the midline of L5. **(B)** Processed PDFF maps of paraspinal muscles; **(C)** manual segmentation of paraspinal muscles, multifidus (red) and erector spinae (blue).

All raw MR images were processed on a commercially available workstation (Advantage Windows 4.6, GE Medical Systems, USA). The degeneration degree of IVD at L4/5 and L5/S1 was assessed by two blinded experienced radiologists according to Pfirrmann grading system (I-V) by MRI T2 (37). The Pfirrmann grading system was divided into five grades to evaluate the homogeneity of intervertebral disc structure, signal strength, discrimination between nucleus and anulus, and disc height. When there was a disagreement, both radiologists discussed to achieve a consensus. Muscle cross-sectional area (CSA) and PDFF values of the bilateral paraspinal muscles were obtained on a region of interest (ROI) basis at the central level of L4/5 and L5/S1. The CSA and PDFF of the paraspinal muscles were measured at two-disc levels for each participant. The two radiologists manually delineated the shape of the bilateral multifidus and erector spinae (**Figure 1**). The muscle CSA was measured by manually delineating the ROI on the axial T2 images, then the same ROI was automatically copied by the workstation to the fat fraction map to obtain the PDFF value. The average of the two measurements was calculated and used for later analysis.

### Statistical Analysis

SPSS 22.0 was performed for the statistical analysis. Mean ± SD was used to express data. Comparisons between patients with CLBP and healthy subjects were determined using the independent-sample t-test. Pearsons correlations and Spearman’s rank correlations were computed between paraspinal muscles CSA, PDFF and Pfirrmann grade, ODI, VSA. One-way analysis of variance (ANOVA) was employed for the comparisons among multiple groups, and Tukey’s multiple comparisons test was utilized for the *post hoc* test after ANOVA. Analysis of covariance was used with age as a covariate to ensure that there was no effect of age on the differences of muscle PDFF. The Cochran-Armitage trend test was used between Pfirrmann grade and other variables. A *p*-value <0.05 was reported statistically significant.

## Result

### Comparison of CSA and PDFF in the Paraspinal Muscle Between Healthy Subjects and Patients With CLBP

**Figure 2:**
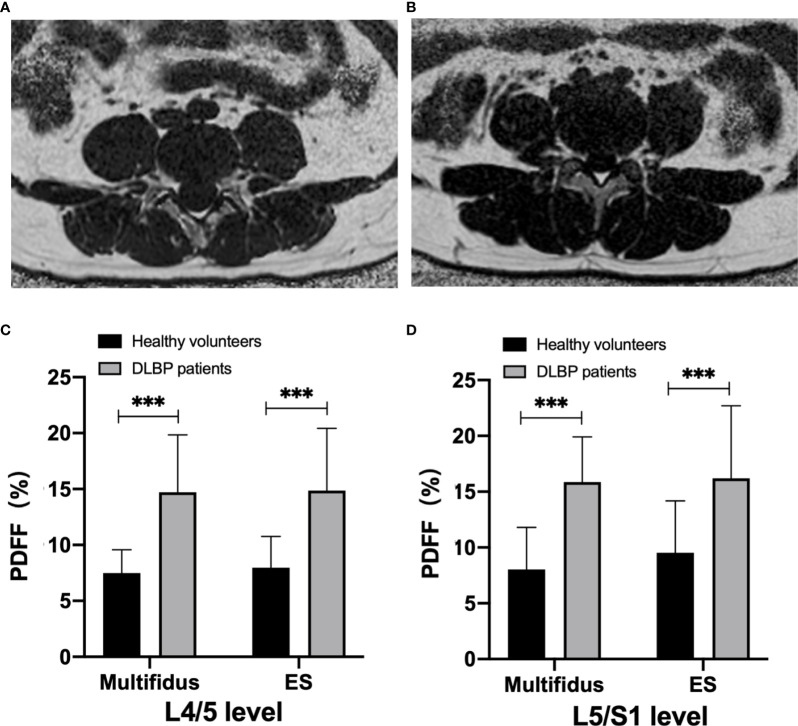
MRI PDFF of lumbar paraspinal muscle in patients with CLBP. **(A)** Patient with CLBP, Male, 29 years old, CLBP for 9 years, PDFF _left multifidus_ = 10.9%, PDFF _right multifidus_ =11.9%, PDFF _left erector spinae_ =10.8%, PDFF _right erector spinae_ = 10.1%. **(B)** Healthy Volunteer, male, 30 years old, PDFF _left multifidus_= 7.2%, PDFF _right multifidus_ = 7.1%, PDFF _left erector spinae_ = 5.1%, PDFF _right erector spinae_ = 4.8%. **(C, D)** Bar chart of paraspinal muscle PDFF at L4/5 and L5/S1 levels. Data are reported as mean ± standard deviation of mean. ***p < 0.001.

There were no differences in gender, age, height, weight, and BMI between healthy subjects and CLBP patients (**Table 1**). The inter-observer agreement of measured CSA and PDFF between two radiologists was good (ICC=0.964, *p*<0.001). The CSAs of the multifidus and erector spinae of CLBP were smaller than those of healthy subjects, but the difference was statistically significant only in the multifidus at the L4/5 level (*p*<0.05, **Table 1**). The PDFF maps showed that the paraspinal muscle PDFFs were increased in patients with CLBP (**Figure 2**). At the level of L4/5 and L5/S1, the multifidus and erector spinae PDFF of CLBP patients were significantly increased than those of healthy subjects, and the differences were statistically significant (all *p <*0.05, **Figure 2**).

### Correlation Between Paraspinal Muscles CSA, PDFF and Pfirrmann Grade of IVDs

**Figure 3:**
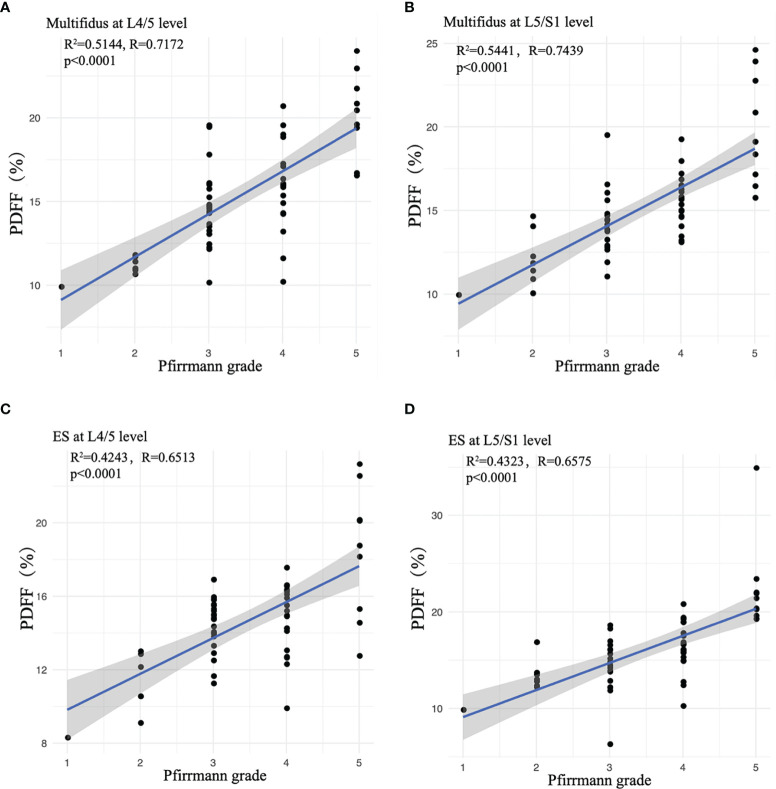
Correlation between age-adjusted PDFF of paraspinal muscles and Pfirrmann grade. **(A)** Multifidus at L4/5 level; **(B)** Multifidus at L5/S1 level; **(C)** ES at L4/5 level; **(D)** ES at L5/S1 level. ES, erector spinae. Spearman’s Rank-Order Correlation was used.

**Figure 4:**
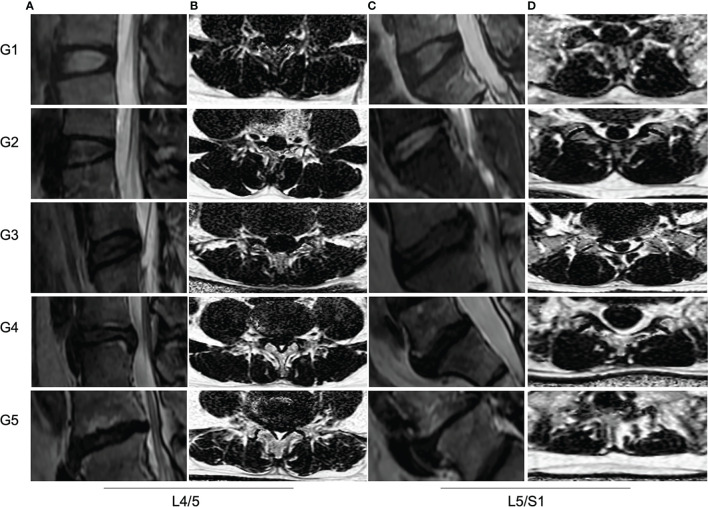
MRI PDFF of lumbar paraspinal muscles at different Pfirrmann grade of IVDs degeneration. **(A, C)** Sagittal T2 images of IVDs at L4/5 and L5/S1 levels. The Pfirrmann grade of IVDs degeneration from left to right are for G1, G2 G3, G4 and G5, respectively. **(B, D)** The PDFF of paraspinal muscles at L4/5 and L5/S1 levels. The mean PDFF at the L4/5 from up to bottom are for 9.1%, 10.5%, 14.2%, 16.3% and 22.3%, respectively. The mean PDFF at L5/S1 from up to bottom are for 10.8%, 12.1%, 14.0%, 16.5% and 24.0%, respectively.

The multifidus CSA was weakly correlated to Pfirrmann grade of IVD degeneration (r=-0.265, *p* =0.004), but there was no significant correlation between the CSA of erector spinae and the Pfirrmann grade (r=-0.305, *p* =0.708). With the increase of Pfirrmann grade of IVDD, the PDFF values of the multifidus and erector spinae in CLBP patients gradually increased, in the order of Grade V>Grade IV>Grade III>Grade II>Grade I (**Table 3**). In the multifidus muscle, the PDFFs of Grade V and Grade IV were higher than that of Grade III and Grade II, and the difference was statistically significant (*p*<0.05, **Table 3**). There were differences in the age of CLBP patients with different Pfirrmann grades, but there was no statistically significant difference in BMI among the groups. After adjusting for age, comparing the multifidus and erector spinae PDFF among different Pfirrmann grades, the results showed that the PDFF of the high-grade Pfirrmann grade were higher than that of the low-grade Pfirrmann grade (**Table 3**). **Figures 3** and **4** show the relationships between multifidus and erector spinae age-adjusted PDFF with Pfirrmann grade at the L4/5 and L5/S1 levels. There was a significant correlation (r = 0.717 and 0.744, all *p* < 0.05) between PDFF of MF and Pfirrmann grade at the IVD levels. In addition, the correlation between erector spinae PDFFs and Pfirrmann grade was lower than that of multifidus (r=0.651 and 0.658, all *p <*0.05). The Pfirrmann grade of IVD degeneration in the control group, 5, 44, 13, 2, 0 discs had Grade I- V, respectively.

**Table 3:** Differences in PDFF and age-adjusted PDFF values of paraspinal muscles between different Pfirrmann grades of two intervertebral discs in CLBP patients $\left(\bar{x}\pm s\right)$ .

Pfirrmann grade	Age	BMI	Multifidus PDFF	Erector spinae PDFF	Multifidus PDFF (adjusted age)	Erector spinae PDFF (adjusted age)
Grade I	23.0 ± 2.8	22.1 ± 1.3	9.9 ± 0.0	9.1 ± 1.1	9.9 ± 0.0	9.1 ± 1.1
Grade II	31.6 ± 6.6	21.8 ± 1.5	11.3 ± 2.1	13.2 ± 4.52	11.2 ± 2.7	14.2 ± 5.5
Grade III	43.3 ± 10.7	22.8 ± 1.4	13.8 ± 3.7^*^	15.3 ± 4.9^*^	13.7 ± 3.9	15.3 ± 5.2
Grade IV	50.6 ± 13.7	22.4 ± 1.9	15.5 ± 4.8^*^	16.5 ± 6.6^*^	15.3 ± 5.0^*^	16.5 ± 7.0^*^
Grade V	53.6 ± 10.1	22.3 ± 1.7	18.4 ± 4.8^*^	19.6 ± 7.2^*^	17.5 ± 5.2^*^	19.0 ± 8.3^*^
*p for trend*	0.000	0.651	0.001	0.008	–	–

^*^Compared with Grade I, p < 0.05.

### Correlation Between Paraspinal Muscles CSA, PDFF and the ODI, VSA of Patients With CLBP

**Table 4** shows the overall relationships between CSA, PDFF of paraspinal muscles and ODI, VSA of CLBP patients. There was a moderate correlation between PDFF, ODI and VSA, and higher than CSA.

**Table 4:** Correlation analysis between CSA, PDFF of paraspinal muscles and ODI, VSA of CLBP patients.

Measurement		ODI			VAS		
		*r*	*p*	95%CI	*r*	*p*	95%CI
CSA							
	MF	-0.257*	0.043	-0.480, 0.026	-0.225	0.102	-0.464, 0.045
	ES	-0.198	0.152	-0.442, 0.074	-0.180	0.214	-0.483, 0.042
PDFF							
	MF	0.437*	0.034	0.023, 0.52	0.368*	0.054	-0.004, 0.505
	ES	0.3134*	0.035	0.021, 0.52	0.379*	0.055	0.119, 0.591

CSA, cross-sectional area; PDFF, proton density fat fraction; MF, multifidus; ES, erector spinae. ODI, oswestry disability index; VAS, visual analog scale. *p < 0.05.

### Analysis of the Difference of CSA and PDFF Regarding Sex and Age

**Figure 5:**
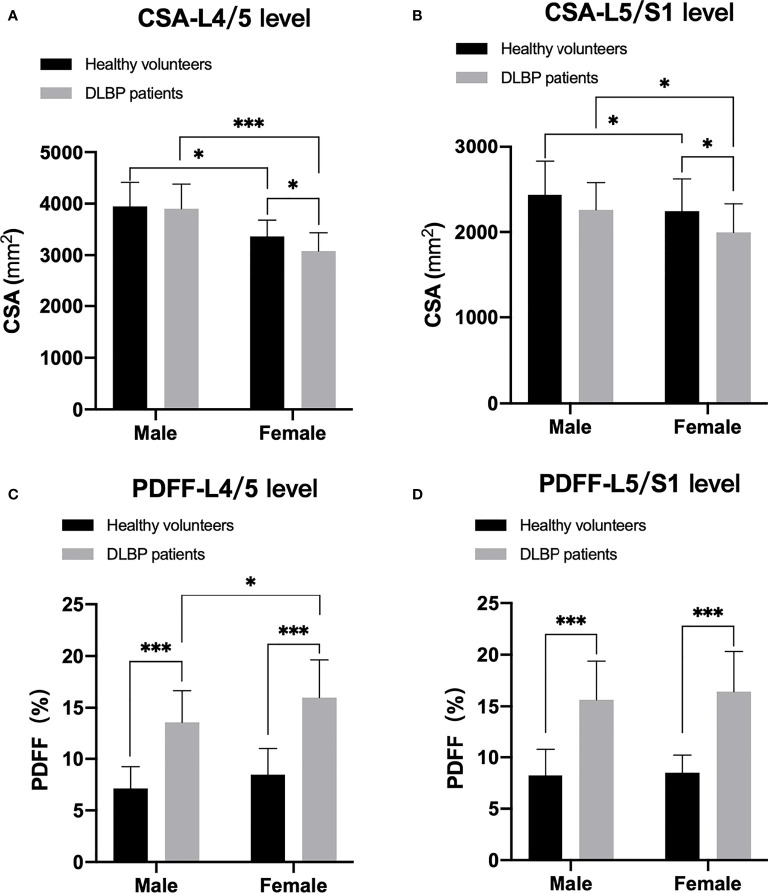
Difference of CSA and PDFF regarding sex. **(A)** CSA at L4/5 level; **(B)** CSA at L5/S1 level; **(C)** PDFF at L4/5 level; **(D)** PDFF at L5/S1 level. *p < 0.05, ***p < 0.001.

**Figure 6:**
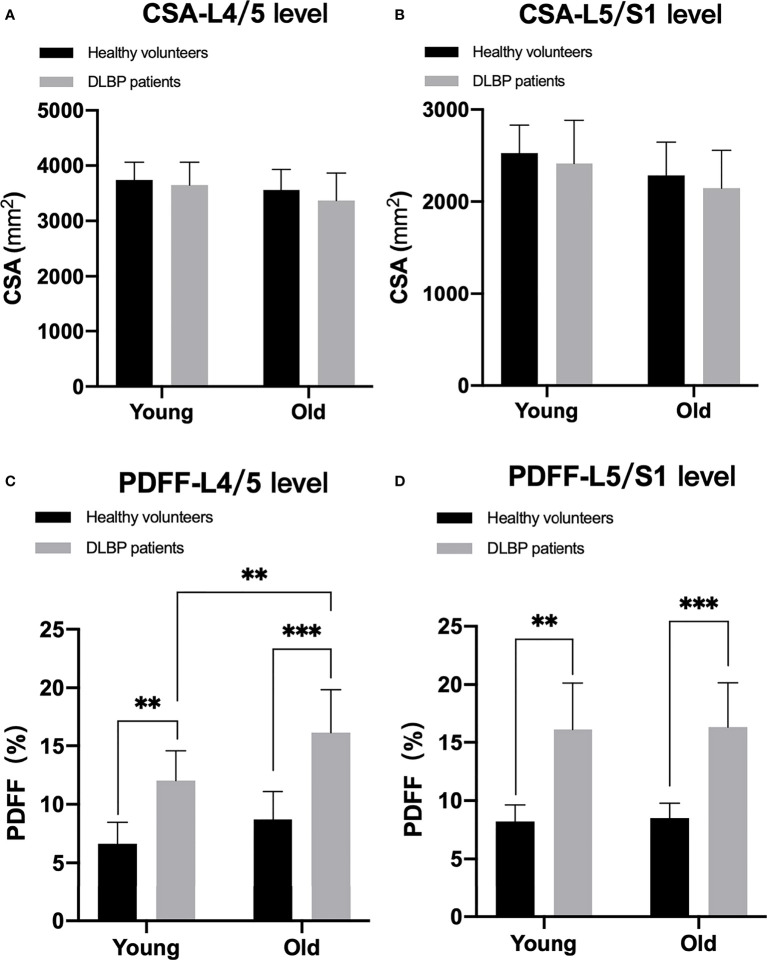
Difference of CSA and PDFF regarding age. **(A)** CSA at L4/5 level; **(B)** CSA at L5/S1 level; **(C)** PDFF at L4/5 level; **(D)** PDFF at L5/S1 level. **p< 0.05, ***p < 0.001.

In healthy subjects and CLBP patients, male paraspinal muscle CSAs at the L4/5 and L5/S1 were larger than females, and the difference was statistically significant (**Figure 5**, all *p <*0.05). In addition, the CSAs of paraspinal muscles in female CLBP patients were lower than that of the healthy subjects, and the difference was statistically significant (**Figure 5**, all *p <*0.05). Regardless of male or female, the PDFFs of the paraspinal muscles showed a significant increase in the CLBP patients. And female CLBP patients were higher than males in the PDFFs at the level of L4/5 (**Figure 5**, all *p*<0.05). The paraspinal muscle CSAs of the old were slightly smaller than that of the young in the both healthy subjects and CLBP patients, but the difference was not statistically significant(**Figure 6**, all *p*>0.05). Whether the old group or young group, CLBP patients were significantly higher than healthy subjects in the PDFFs of the paraspinal muscles (**Figure 6**, all *p*<0.05). And the PDFFs of the paraspinal muscles of the elderly CLBP patients were higher than that of the young at the level of L4/5 (**Figure 6**, *p*<0.05).

### Multiple Linear Regression Analysis

**Table 5** shows the multiple linear regression analysis of paraspinal muscle PDFFs. Age, gender, and Pfirrmann grade of IVDs were independent factors of multifidus FF value (*p <*0.05), and Pfirrmann grade of IVDs was an independent factor of erector spinae PDFF value (*p <*0.05).

**Table 5:** Multiple linear regression analysis of PDFF of paraspinal muscles.

Factor	PDFF of multifidus			PDFF of erector spinae		
	Standardized β	95% CI	*p*	Standardized β	95% CI	*p*
Age	0.233	(0.054, 0.412)	0.011	0.192	(-0.004, 0.388)	0.055
Gender	0.193	(0.033, 0.354)	0.019	0.107	(-0.069, 0.283)	0.232
Pfirrmann grading	0.322	(0.141, 0.503)	0.001	0.215	(0.017, 0.413)	0.034

Standardized β is the standardized regression coefficient.

## Discussion

Using quantitative MR imaging, our study showed that the paraspinal muscles atrophy and fat content increase in patients with CLBP compared to healthy subjects. And a significant correlation was observed between the degeneration of the lumbar spine IVD and the PDFF of adjacent paraspinal muscle in patients with CLBP. Furthermore, sex and age were also independently associated with the paraspinal muscle fat.

Compared with healthy subjects, the CSA of the paraspinal muscles of CLBP patients were decreased only in the multifidus muscle at the L4/5 level. Although the muscle CSA is the most studied, paraspinal muscle atrophy in CLBP remains controversial (38–40). Barker et al. used conventional MRI to compare the multifidus CSA of patients with unilateral pain CLBP, and the results showed that the multifidus CSA of the painful side was lower than that of the asymptomatic side (41). The strength of the paraspinal muscles measured during maximal isometric trunk flexion and trunk extension contractions is decreased in patients with CLBP (42). Some studies have found that muscle CSA was reduced but not significant (38). This may be related to changes in the composition of muscles. When muscle tissue is atrophy, fat infiltration occurs in the paraspinal muscles (29, 43). To some extent, due to the filling and replacement of adipose tissue, the overall muscle CSA has not changed significantly. Our findings in this study further support this view.

In our study, compared with the control group, the PDFF of the paraspinal muscles of CLBP patients was significantly increased. Yanik et al. quantified the fat content of multifidus muscle in patients with CLBP and asymptomatic subjects by conventional MRI, and the results were consistent with this study (33, 44, 45). On the basis of the previous research, we manually delineated the edge of the muscle as the ROI, and further applied the multi-echo Dixon method, which corrected the main magnetic field inhomogeneity effect, T2* effect, T1 effect and other confounding factors, in order to make the quantification of paraspinal muscle fat content is more accurate, better repeatability and reliability (46–48). In order to minimize the possible impact of the slightly differences in spatial resolutions of T2 and PDFF images, we selected the layers at the center of the L4/5 and L5/S1 intervertebral discs as much as possible to delineate the ROI of the paraspinal muscles. In addition, we artificially removed cases with obvious motion artifacts. In this study, we further found the correlation between PDFF and IVD degeneration is higher than correlations between CSA and IVD degeneration. It indicated that the paraspinal muscles of CLBP patients had muscle tissue atrophy and fat replacement.

Recently, much attention has focused on lumbar IVD degeneration in CLBP patients and it is related to the Oswestry Disability Index (41, 49). Paraspinal muscle may play an important role in elucidating and treating lumbar spine dysfunction and spinal imbalance (50, 51). Indeed, we found significant correlations between IVD degeneration of the lumbar spines and the PDFF of adjacent paraspinal muscles in our cohort. Sato et al. revealed that muscle CSA changes were more correlated with pressure pain sensitivity in CLBP patients (52). This difference may be related to the different pain measurement methods we use. Furthermore, the multifidus muscle is more significantly affected. The anatomical relationships between the multifidus muscle and lumbar spine would be relevant to this interpretation. In the lumbar spine, the multifidus muscle is the most developed and important. The multifidus contributes to side bending (tilting) and rotation (twisting) (53). Compared with the erector spinae, the multifidus muscle is more closely related to the lamina and spinous process (54). And the multifidus is a short muscle which makes it more local and prone to changes at the L4/5 and L5/S1levels. The stability of the spine is reduced after CLBP, and muscle changes may be used as a compensatory strategy to cause long-term paraspinal muscle fatigue. When the muscle is decompensated, it will promote the recurrence or exacerbation of CLBP (55). This underlines the importance of the muscles PDFF in the context of IVD degradation. The increased fat infiltration in the paraspinal muscles may be related to the inflammatory disorders found in the multifidus muscles of patients with degenerative spine (56). In animal experiments, James et al. found an increase in macrophages and TNF in the multifidus muscle of a sheep model of IVD degeneration (57). The increased inflammation is likely to be an important factor in promoting fat infiltration of skeletal muscle (58). At the same time, we found that the level of pain and dysfunction in CLBP patients were higher, but the direct relationship between IVD degeneration, paraspinal muscle remodeling, pain, and dysfunction still needs further exploration.

In this study, we account for the potential effects of sex, age, and BMI on muscle fat. In the normal population, males have a larger CSA of paraspinal muscles and lower fat content than females. This is consistent with the results of previous studies (59). We found that both males and females with CLBP have muscle fat infiltration. And it seems to be more pronounced in females, the underlying mechanism is the decline in muscle performance caused by hormone deficiency after menopause in women (43, 60). Age is an important factor in the fat infiltration of paraspinal muscles. Our results show that paraspinal muscle fat increases with age (59, 61), indicating that paraspinal muscle are gradually deteriorating, even in healthy individuals. Therefore, it is necessary to consider the relationship between paraspinal muscles and spinal degeneration with age as a covariate. The PDFFs of the paraspinal muscles in both young and old CLBP patients were significantly increased, the increase was more significant in the elderly. This may be related to the poorer basic muscle strength and performance of the elderly. Fat infiltration of paravertebral muscles in CLBP patients can only be supported if age and sex effects are fully clear. In this study, through the ODI scale test, we observed that the activity level of CLBP patients was reduced. Hodges and Goubert et al. believes that pain leads to disuse muscle atrophy caused by reduced multifidus muscle activity (45, 62). But several previous researches conclude that as to the assumption that patients with CLBP suffer from disuse and physical deconditioning empirical evidence is still lacking (63, 64). In future studies, the activity level of CLBP patients deserves further consideration.

There are several limitations of this study. First, cross-sectional design with relatively small subjects is considered the main limitation. Subsequent longitudinal cohort studies are warranted to further investigate and confirm the relationship between IVD degeneration and paraspinal muscle fat infiltration. Furthermore, the current intervertebral degeneration grading system is qualitative and subjective, and a quantitative method is a better choice. Moreover, the L4/5 and L5/S1 IVDs are regarded as the level of interest because they are the most degradable levels. However, it is unclear whether the paraspinal muscles at the level of the L1-4 IVDs have changed. The post-processing software used in this study can obtain the PDFF of muscle, but not the PDFF of muscle tissue. So, the intermuscular and intramuscular fat cannot be completely distinguished in this study. At last, it will be interesting to further explore the distribution of fat in the paraspinal muscles and clearly quantify the levels of lipids within and outside muscle cells.

Using quantitative MRI to measure CSA and PDFF, this study confirmed the changes in CSA and PDFF of the paraspinal muscles in CLBP patients and found a significant correlation between lumbar IVD degeneration and the PDFF of paraspinal muscles. Sex and age are important factors also considered influencing factors for the paraspinal muscles in CLBP patients. Our findings clearly highlighted the assessment of fat content within paraspinal muscles in CLBP patients and might trigger a paradigm shift in the intervention strategy to CLBP. Paraspinal muscle fat infiltration should also be evaluated as treatment outcome, and its use as a treatment endpoint for therapies should be further investigated.

## Data Availability Statement

The original contributions presented in the study are included in the article/supplementary material. Further inquiries can be directed to the corresponding authors.

## Ethics Statement

The studies involving human participants were reviewed and approved by Kunming Medical University. The patients/participants provided their written informed consent to participate in this study. Written informed consent was obtained from the individual(s) for the publication of any potentially identifiable images or data included in this article.

## Author Contributions

BH and XC: Designed the study and conceived the report. YH and LW: Wrote the draft of the manuscript and revised it critically. XZ, JC, ZZ and YJ: Data acquisition and processing. BH and YH: Analyzed and interpreted the results of MRI. LN: Technical support for MRI scanning. YH, LW and XZ: Statistical analysis, and created the figures and tables. All authors had read and approved the final manuscript.

## Funding

This work is supported by the Applied Basic Research Project of Yunnan Province- Kunming Medical University Joint Fund (202001AY070001-038), Beijing Hospitals Authority Youth Programme (QMS20200402), and Yunnan Provincial Bone and Joint Disease Clinical Medicine Center Project (ZX2019-03-04).

## Conflict of Interest

XZ and LN were employed by GE Healthcare.

The remaining authors declare that the research was conducted in the absence of any commercial or financial relationships that could be construed as a potential conflict of interest.

## Publisher’s Note

All claims expressed in this article are solely those of the authors and do not necessarily represent those of their affiliated organizations, or those of the publisher, the editors and the reviewers. Any product that may be evaluated in this article, or claim that may be made by its manufacturer, is not guaranteed or endorsed by the publisher.
